# Human Motion Retrieval Based on Statistical Learning and Bayesian Fusion

**DOI:** 10.1371/journal.pone.0164610

**Published:** 2016-10-12

**Authors:** Qinkun Xiao, Ren Song

**Affiliations:** Department of Electronic Information Engineering, Xi’an Technological University, Xi’an City, Shaanxi Province, China P.R, 710032; Southwest University, CHINA

## Abstract

A novel motion retrieval approach based on statistical learning and Bayesian fusion is presented. The approach includes two primary stages. (1) In the learning stage, fuzzy clustering is utilized firstly to get the representative frames of motions, and the gesture features of the motions are extracted to build a motion feature database. Based on the motion feature database and statistical learning, the probability distribution function of different motion classes is obtained. (2) In the motion retrieval stage, the query motion feature is extracted firstly according to stage (1). Similarity measurements are then conducted employing a novel method that combines category-based motion similarity distances with similarity distances based on canonical correlation analysis. The two motion distances are fused using Bayesian estimation, and the retrieval results are ranked according to the fused values. The effectiveness of the proposed method is verified experimentally.

## 1 Introduction

In recent years, computer animation has become increasing employed in various applications [1–8]. The application of computer animation to human motion is of particular interest. This has led to a high demand for producing very realistic representations of human movement. Many approaches have been developed to generate human motion data. Motion capture (MoCap) is a well-known method. The increasing availability of MoCap devices has driven the development of large human and object motion databases [8, 9]. However, as the variety of motion data grows, it is a hard thing to search suitable motions satisfying specific requirements. Hence, motion retrieval has recently turned into a main research focus in the field of MoCap animation.

Some motion retrieval approaches have been proposed in the literature, and many of these are modified from the existing audio retrieval methods, such as the dynamic time warping (DTW) method [9,10]. However, the application of DTW to MoCap data typically demonstrates low efficiency due to the large number of attributes and parameters for this type of data. To support indexing and to increase the retrieval performance of DTW, an algorithm based on DTW and uniform scaling (US) has been proposed [11]. However, the method usually cost more time. An extensional method based on the DTW and canonical correlation analysis (CCA), is called as generalized canonical time warping (GCTW), has been proposed for adjusting multi-modal sequences [12]. In addition to the DTW-based methods, other methods seek to match logically similar motions. For example, templates have been developed for presenting motion, and motion search using template matching was proposed [13]. In addition, geometric features have been employed to construct indexing trees using clustering and segmentation, action matching by peak points was proposed [14]. However, these methods cannot distinguish closely matching movements.

Besides above-mentioned, some novel methods are also presented to recognize or retrieve human actions. Jan Baumann *et al*. [15] propose a generic data-driven method for recognizing human full body actions. The approach is online-capable, and works in real time. They extract skeletons from depth sensors and video data. Evaluation results on a freely available motion capture database indicate the action recognition method is effectiveness and superior than frame-based support vector machine approach. Some technical companies, such as Microsoft and HP, also strive to recognize human gestures in real-time using the depth sensors (such as Kinect) [16]. In [17], the gesture recognition problem is translated into a pixel-based classification problem. The deep randomized decision forests are built based on data learning, the recognition can be finished in real-time.

Based on above-mentioned, in the present study, we are interested in retrieving motions from a motion database that are closely similar to a given query motion. The general motivations for the motion retrieval method proposed herein can be described as follows.

(1) As discussed above, the DTW-based retrieval methods indicate better performance than statistical matching methods, but with lower efficiency, so we propose combining DTW-based matching with statistical motion matching for enhanced motion matching performance and efficiency.

**Fig 1:**
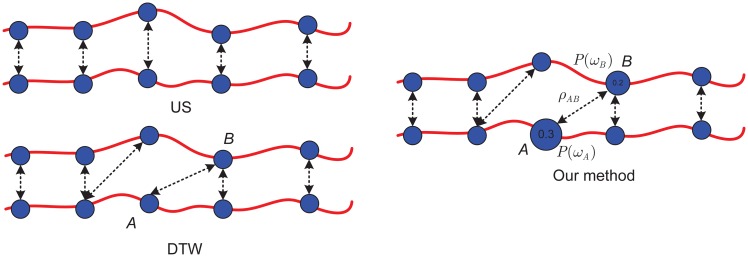
Principle comparison of between US, DTW and our method.

The DTW is an algorithm for measuring similarity between two time series data. The DTW method is based on optimal match calculation between two given sequences with certain restrictions. We compare our idea with the US and DTW in Fig 1, the US is a linear transformation that enlarges or shrinks objects by a scale factor, the US similarity distance calculation is mainly based on frames matching one by one, which is a older sequence matching approach, has been used for motion and multimedia data retrieval [18][19]. The DTW similarity distance calculation is mainly based on optimal match using dynamic programming (DP). However, during matching, some factors, such as weights, probability distributions, and correlations of frames are not considered in DTW calculation. In fact, the factors maybe influence match results greatly. Inspired by that, we consider improving DTW calculation through 3 steps, one is to consider improving DTW distance using canonical correlation analysis, we call it is CCA-based distance, two is to consider improving DTW distance using probability distribution of frames, we call it is class-based distance, the last step, for enhancing match performance further, similar to boosting method, a stronger classifier is obtained by two weaker classifier combination, we use Bayes fusion to put the CCA-based distances and class-based distances together, the fused distances could be more effectiveness.

At the same time, in recent years, for enhancing the sequence matching performance, some improved algorithm based on DTW have been represented. Hsu *et al*. [20] proposed a robust sequence matching method named iterative motion warping (IMW), it is used to find spatio-temporal warping between two action sequences. Shapiro *et al*. [21] decompose action data using independent component analysis, and action data can be separated into some visually meaningful components. Those methods are improved DTW approaches, and usually can handle with certain type of data. In comparison, our method solves a more general sequence matching problem of human motion. In [22], based on constraints from the fundamental matrix, moving trajectories are aligned. In [23], a adopted DTW is proposed based on a view-invariant description for synchronizing human actions. These algorithms is devoted to improve frames alignment technology, it can be seen as further improvement or optimization of the DTW or US methods, hence, those algorithms further increase sequence matching accuracy. However, those methods either focus on optimal spatial relations or focus on temporal correlation, in comparison, our approach can calculate the optimal spatial relations and temporal correlation of action sequences at the same time.

(2) The next, as we known, the representative discriminating features based on optimization usually have better performance than the primitive disorder descriptors, and we therefore translate the redundancy gesture features into discriminating descriptors using clustering [24]. Multivariate statistical learning and a Bayesian fusion methodology [25] are employed to convert motion matching into a transportation problem for accommodating rotation, and local or global scaling. We compare the performance of the proposed algorithm with the performances of the DTW and US methods, and our experimental results demonstrate the promising accuracy and effectiveness of the proposed algorithm.

The remainder of the paper is constructed as follows. The proposed algorithm is described in Section 2. The testing results are shown in Section 3, and Section 4 gives a briefly concludes.

## 2 Retrieval Algorithm

### 2.1 Overview of algorithm

**Fig 2:**
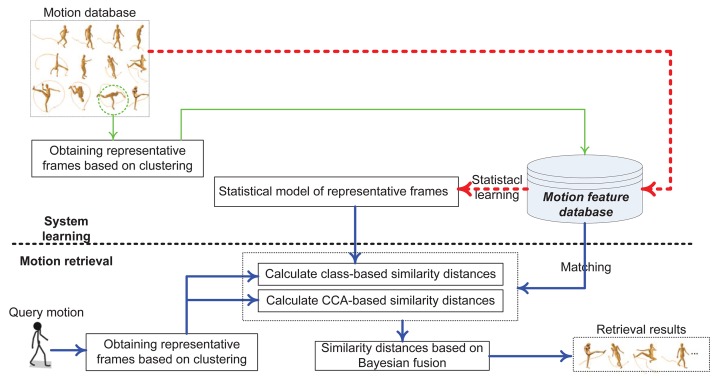
Overview of the proposed algorithm.

The proposed algorithm is schematically illustrated in Fig 2, where the algorithm is divided into two stages: system learning and motion retrieval.

In the system learning stage, a motion database is constructed firstly, and is comprised of distinguishable motions. Secondly, as illustrated in Fig 2, motion features are extracted according to the two steps: (1) obtain representative frame based on clustering, and (2) statistical model of representative frames. In the motion retrieval stage, the query motion feature is built based on above-mentioned steps. The motion matching includes steps are illustrated in Fig 2 too. The steps include: (1) calculate class-based similarity distances, (2) calculate CCA-based similarity distances, and (3) calculate similarity distances based on Bayesian fusion. The details are described as follows.

### 2.2 System learning

#### 2.2.1 Obtain representative frame based on clustering

Given a motion sequence {**F**_i_}_i = 1:n_, where the n is the number of frames, the representative frames of the motion are generated using the fuzzy c-means (FCM) clustering approach. To compute distances between two frames, the quaternion [11] is used to present body gesture. Let the **F**_i_ be a motion descriptor in the i-th frame. The distance between **F**_1_ and **F**_2_ is computed as: d(**F**_1_,**F**_2_) = (**F**_1_−**F**_2_)(**F**_1_−**F**_2_)^T^. If we use clustering method to generate c clustering centers, the frames with the shortest distances to clustering centers can be selected as the representative frames. Let representative frames be **RF** = {*rf*_*k*_}_*k* = 1:*c*_, where the *rf*_*k*_ is gesture feature corresponding to the *k*-th clustering center. Based on the FCM, each motion has different the number of clustering centers. At the same time, obtained representative frames have different weights corresponding to different cluster sizes. Let weight matrix be **w** = {*w*_*k*_}_*k* = 1:*c*_, and the *w*_*k*_ is defined as: *w*_*k*_ = *C*(*k*)/*n*, where the *C*(*k*) is the number of frames in *k*-th clustering group.

In this paper, we use FCM clustering to obtain the representation frames, the FCM parameters include iterations (denoted as *l*_*max*_), membership matrix weighted index (denoted as *w*_*U*_), minimum variation of membership (denoted as *ε*), we set *w*_*U*_ = 2, *l*_*max*_ = 100, *ε* = 1e-5, and number of cluster center (denoted as *k*). The *k* is important for retaining motion clips information, we set *k* = round(*n*_fram_/*α*), where round(.) is round function, *n*_fram_ is the total frames number of motion clip, and *α* is clustering coefficient, in this paper, we set *α* according to motion dataset scale and retrieval effectiveness, for example, in HDM cut database, due to there are many shorter motion sequence data, we set *α* = 45, assume a 221 frames clip, the number of clustering center is: *k* = round(221/45) = 5, the longer clips, the more centers. In general, the more centers are with the higher retrieval accuracy and the more time cost, in section 3.3, we will discuss influence between this parameter and retrieval performance.

#### 2.2.2 Statistical model of representative frames

According to above-mentioned, a motion is presented using representative frames and the corresponding weight matrix. In this paper, we assume that the **RF** = {*rf*_*k*_}_*k* = 1:*c*_ is the Gaussian distribution, and the purpose of statistical learning is to obtain the relevant parameters. Let datasets be **D** = {*ω*_*j*_}_*j* = 1:*p*_ where *ω*_*j*_ is *j*-th semantic groups in motion datasets, at the same time, assume the *ω*_*j*_ includes *n*1 motion descriptors, let **RF**_*i*_^*j*^ be the *i*-th motion descriptor in *ω*_*j*_, we have: *ω*_*j*_ = {**RF**_*i*_^*j*^}_*i* = 1:*n*1_, where the **RF**_*i*_^*j*^ = {*rf*_*k*_^*i*^}_*k* = 1:*ci*_, at the same time, the weight matrix is **W**_*j*_ = {**w**_*i*_^*j*^}_*i* = 1:*n*1_, where the **w**_*i*_^*j*^ = {**w**_*k*_^*i*^}_*k* = 1:*ci*_. Suppose gesture feature *rf*_*k*_^*i*^ is multivariate normal distribution, the probability density function of the *rf*_*k*_^*i*^ is: $p(rf)=\mathbb{N}({\hat{\mu }}_{\omega_{j}\in rf},{\hat{\Sigma }}_{\omega_{j}\in rf})$, where the parameters are:

$${\hat{\mu }}_{\omega_{j}}=\frac{\sum_{i=1}^{n1}\sum_{k=1}^{ci}w_{k}^{i}rf_{k}^{i}}{\sum_{i=1}^{n1}\sum_{k=1}^{ci}k}$$ (1)

$${\hat{\Sigma }}_{\omega_{j}}=\frac{\sum_{i=1}^{n1}\sum_{k=1}^{ci}(w_{k}^{i}\cdot rf_{k}^{i}-{\hat{\mu }}_{\omega_{j}}){\left(w_{k}^{i}\cdot rf_{k}^{i}-\mu_{\omega_{j}}\right)}^{T}}{\sum_{i=1}^{n1}\sum_{k=1}^{ci}k}$$ (2)

### 2.3 Motion retrieval

#### 2.3.1 Calculate class-based similarity distances

Similar to system learning, the query motion descriptor is **RF**^*q*^ = {*rf*_*k*_^*q*^}_*k* = 1:*cq*_, which corresponding to **w**^*q*^ = {*w*_*k*_^*q*^}_*k* = 1:*cq*_. There are many similarity measure methods to calculate the class-based distance, such as, Minkowski distance or Manhattan distance and Mahalanobis Distance, and so on. According to the above-mentioned, if statistical property is considered fully, the distance between query motion and semantic group *ω*_*j*_ is:

$$d(RF^{q},\omega_{j})=\frac{\sum_{k=1}^{cq}(w_{k}^{q}\cdot rf_{k}^{q}-{\hat{\mu }}_{\omega_{j}}){\hat{\sum }}_{\omega_{j}}^{-1}{\left(w_{k}^{q}\cdot rf_{k}^{q}-\mu_{\omega_{j}}\right)}^{T}}{cq}$$ (3)

Meanwhile, the DTW [10] distance between the **RF**^*q*^ and the **RF**^*i*^ is *d*_*DTW*_(**RF**^*q*^, **RF**^*i*^), hence, the class-based distance is:

$$d_{class}(RF^{q},RF^{i})=d_{DTW}(RF^{q},RF^{i})\cdot d(RF^{q},\omega_{j})$$ (4)

where the **RF**^*i*^∈*ω*_*j*_.

#### 2.3.2 Calculate CCA-based similarity distances

We consider two motions: **RF**^*q*^ = {*rf*_*k*_^*q*^}_*k* = 1:c*q*_ and **RF**^*i*^ = {*rf*_*k*_^*i*^}_*k* = 1:*ci*_. Based on CCA relevant theories [12], we can calculate the canonical correlation coefficient between the **RF**^*q*^ and the **RF**^*i*^ as:

$$\rho_{RF^{q},RF^{i}}=\frac{\text{cov}(A\cdot RF^{q},B\cdot RF^{i})}{\sqrt{\text{var}(A\cdot RF^{q})\text{var}(B\cdot RF^{i})}}$$ (5)

where **A** and **B** are parameters. Based on parameters optimization, the optimal $\rho_{RF^{q},RF^{i}}$ is got, which is denoted as $\rho_{RF^{q},RF^{i}}^{*}$. Meanwhile, we also compute DTW distance between the **RF**^*q*^ and the **RF**^*i*^. This provides the CCA-based distance:

$$d_{CCA}(RF^{q},RF^{i})=d_{DTW}(RF^{q},RF^{i})\cdot \rho_{RF^{q},RF^{i}}^{*}$$ (6)

#### 2.3.3 Similarity distances based on Bayesian fusion

**Fig 3:**
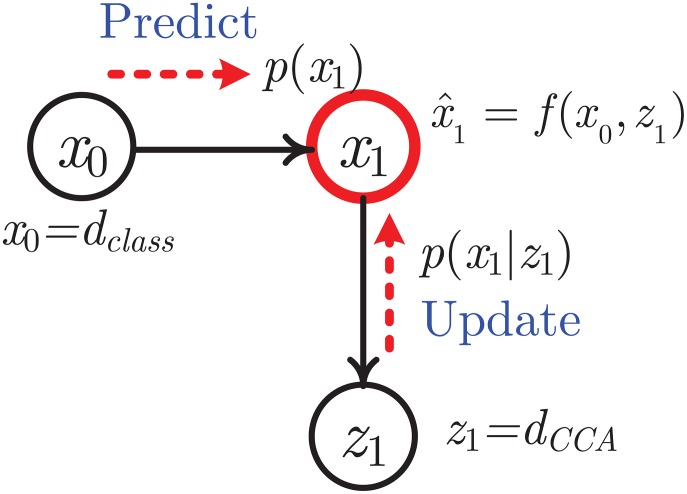
Illustration of similarity distances calculation based on Bayesian fusion.

The *d*_*class*_ and the *d*_*CCA*_ are fused based on a Bayesian graphical model. The final fused similarity would be used for motion retrieval to further enhance retrieval precision. We construct graph model shown in Fig 3. Let *x*_0_ = *d*_*class*_ be value of prior knowledge, and let *z*_1_ = *d*_*CCA*_ be measurement data. The fused estimation value is ${\hat{x}}_{1}=f\left(x_{0},z_{1}\right)$.

For obtaining optimal fusion values, according to Bayesian probability theory, we first predict *x*_1_ using the prior state *x*_0_, according to probability model, we have: *p*(*x*_1_) = *p*(*x*_1_|*x*_0_)*p*(*x*_0_). Meanwhile, the predicted *x*_1_ is updated using the measurement *z*_1_, according to probability model, we have: *p*(*x*_1_|*z*_1_) = *αp*(*z*_1_|*x*_1_)*p*(*x*_1_). The detail is described as follows.

Suppose all variables in Fig 3 are Gaussian distributions, and we have:

$$p(x_{0})=\mathbb{N}(\mu_{0},\sigma_{0}^{2})=\alpha e^{-\frac{1}{2}\left(\frac{{\left(x-\mu_{0}\right)}^{2}}{\sigma_{0}^{2}}\right)}$$ (7)

where *μ*_0_ and *σ*_0_^2^ are expectation and variance of the *x*_0_, respectively, and parameters are obtained through prior knowledge and data learning. Suppose conditional probability distribution *p*(*x*_1_|*x*_0_) is:

$$p(x_{1}|x_{0})=\mathbb{N}(x_{0},\sigma_{x}^{2})=\alpha e^{-\frac{1}{2}\left(\frac{{\left(x_{1}-x_{0}\right)}^{2}}{\sigma_{x}^{2}}\right)}$$ (8)

where *σ*_*x*_^2^ is the variance of prediction *x*_1_. The parameters are obtained through prior knowledge and data learning. According to Bayes' theorem, the following holds.

$$\begin{array}{l} p(x_{1})=p(x_{1}|x_{0})p(x_{0})\\ =\alpha \int_{-\infty }^{+\infty }e^{-\frac{1}{2}\left(\frac{{\left(x-x_{0}\right)}^{2}}{\sigma_{x}^{2}}\right)}e^{-\frac{1}{2}\left(\frac{{\left(x_{0}-\mu_{0}\right)}^{2}}{\sigma_{0}^{2}}\right)}dx_{0}\\ =\alpha \int_{-\infty }^{+\infty }e^{-\frac{1}{2}\left(\left(\frac{1}{\sigma_{x}^{2}}+\frac{1}{\sigma_{0}^{2}}\right)x_{0}^{2}+\left(\frac{-2x_{1}}{\sigma_{x}^{2}}+\frac{-2\mu_{0}}{\sigma_{0}^{2}}\right)x+\left(\frac{x_{1}^{2}}{\sigma_{x}^{2}}+\frac{\mu_{0}^{2}}{\sigma_{0}^{2}}\right)\right)}dx_{0} \end{array}$$ (9)

If let $a=\left(\frac{1}{\sigma_{x}^{2}}+\frac{1}{\sigma_{0}^{2}}\right)$, $b=\left(\frac{-2x_{1}}{\sigma_{x}^{2}}+\frac{-2\mu_{0}}{\sigma_{0}^{2}}\right)$ and $c=\left(\frac{x_{1}^{2}}{\sigma_{x}^{2}}+\frac{\mu_{0}^{2}}{\sigma_{0}^{2}}\right)$, we have:

$$\begin{array}{l} p(x_{1})=\alpha e^{-\frac{1}{2}\left(c-\frac{b^{2}}{4a}\right)}\int_{-\infty }^{+\infty }e^{-\frac{1}{2}a{\left(x_{0}-\frac{-b}{2a}\right)}^{2}}dx_{0}\\ =\alpha e^{-\frac{1}{2}\left(c-\frac{b^{2}}{4a}\right)}=\alpha e^{-\frac{1}{2}\left(\frac{{\left(x_{0}-\mu_{0}\right)}^{2}}{\sigma_{0}^{2}+\sigma_{x}^{2}}\right)} \end{array}$$ (10)

Here, $\int_{-\infty }^{+\infty }e^{-\frac{1}{2}a{\left(x_{0}-\frac{-b}{2a}\right)}^{2}}dx_{0}=1$.

The next, the *x*_1_ can be updated by measurement *z*_1_:

$$\begin{array}{l} p(x_{1}|z_{1})=\alpha p(z_{1}|x_{1})p(x_{1})\\ =\alpha e^{-\frac{1}{2}\left(\frac{{\left(z_{1}-x_{1}\right)}^{2}}{\sigma_{z}^{2}}\right)}e^{-\frac{1}{2}\left(\frac{{\left(x_{1}-\mu_{0}\right)}^{2}}{\sigma_{z}^{2}+\sigma_{x}^{2}}\right)} \end{array}$$ (11)

Employing the method of completing the square yields:

$$p(x_{1}|z_{1})=\alpha e^{-\frac{1}{2}\left(\frac{{\left(x_{1}-\frac{(\sigma_{0}^{2}+\sigma_{x}^{2})z_{1}+\sigma_{z}^{2}\mu_{0}}{\sigma_{0}^{2}+\sigma_{x}^{2}+\sigma_{z}^{2}}\right)}^{2}}{\left(\frac{(\sigma_{0}^{2}+\sigma_{x}^{2})\sigma_{z}^{2}}{\sigma_{0}^{2}+\sigma_{x}^{2}+\sigma_{z}^{2}}\right)}\right)}=\alpha e^{-\frac{1}{2}\left(\frac{{\left(x_{1}-\mu_{1}\right)}^{2}}{\sigma_{1}^{2}}\right)}$$ (12)

Lastly, based on *E*(*x*_0_) = *μ*_0_ = *d*_*class*_ and *z*_1_ = *d*_*CCA*_, let $E(x_{1})=\mu_{1}={\hat{x}}_{1}$, and using Bayesian fusion [25], the final fused distance between **RF**^q^ and **RF**^*i*^ is:

$$\begin{array}{l} d_{new}(RF^{q},RF^{i})={\hat{x}}_{1}=f(x_{0},z_{1})=f(d_{class},d_{CCA})\\ =\frac{(\sigma_{0}^{2}+\sigma_{x}^{2})z_{1}+\sigma_{z}^{2}x_{0}}{\sigma_{0}^{2}+\sigma_{x}^{2}+\sigma_{z}^{2}}=\frac{\left(\sigma_{0}^{2}+\sigma_{x}^{2})d_{CCA}(RF^{q},RF^{i})+\sigma_{z}^{2}d_{class}(RF^{q},RF^{i}\right)}{\sigma_{0}^{2}+\sigma_{x}^{2}+\sigma_{z}^{2}} \end{array}$$ (13)

**Fig 4:**
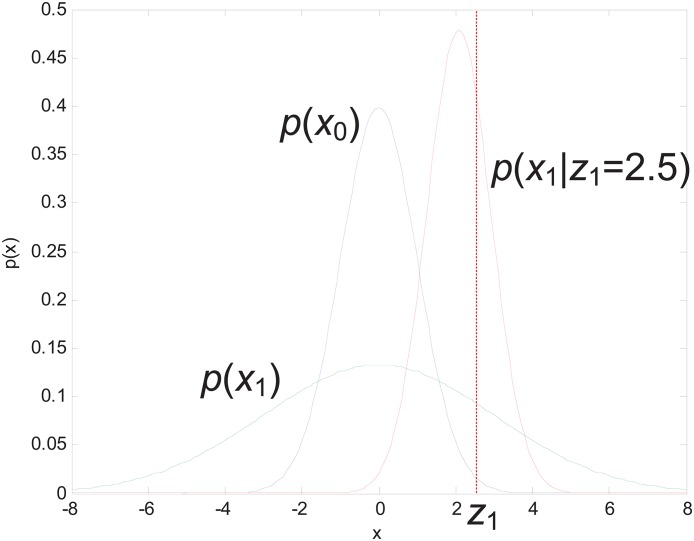
Example for Bayesian fusion calculation.

A simple Bayesian fusion example is shown in Fig 4, where $p(x_{0})=\mathbb{N}(\mu_{0},\sigma_{0}^{2})$, $p(x_{1})=\mathbb{N}(\mu_{0},\sigma_{0}^{2}+\sigma_{x}^{2})$ and $p(x_{1}|z_{1})=\mathbb{N}\left(\frac{(\sigma_{0}^{2}+\sigma_{x}^{2})z_{1}+\sigma_{z}^{2}\mu_{0}}{\sigma_{0}^{2}+\sigma_{x}^{2}+\sigma_{z}^{2}},\frac{(\sigma_{0}^{2}+\sigma_{x}^{2})\sigma_{z}^{2}}{\sigma_{0}^{2}+\sigma_{x}^{2}+\sigma_{z}^{2}}\right)$, to set *σ*_0_ = 1, *σ*_*x*_ = 2, *σ*_*z*_ = 1, *μ*_0_ = 0, and *z*_1_ = 2.5. The final updated value is *x*_1_ = 2.1.

Finally, the matching results are ranked according to the *d*_*new*_, and the top-*p* motions (*p* is typically set to 20) with the smallest fusion distance values are returned to the user.

## 3 Experiments

Some experiments have been conducted to verify performance of our proposed retrieval approach. Two famous motion databases, Carnegie Mellon University (CMU) motion capture database [9] and HDM05 motion cut database [8], are used in our experiments. The first motion database includes roughly 2500 different motion clips and roughly 100 categories, the second motion database includes roughly 2400 different motion clips and roughly 100 categories. Each of the original motion clips typically contained more than a single activity. The testing objective is to search the best matched motion clips in motion database in term of the given query motion. For comparison with the proposed statistical learning and Bayesian fusion (SLBF) method, we have also implemented the DTW [10] and the US method [11]. All tests are evaluated on a PC with a Pentium 6 GHz CPU and 2 GB RAM.

### 3.1 Performance of distance matching

The proposed CCA-based and class-based similarity distance matching is the fundamental of the SLBF, we first test performance of the CCA-based matching, the class-based matching and SLBF-based matching, respectively. The performance comparisons experiments between the DTW and the 3 kind of distances are conducted. We use retrieval precision to evaluate the performance of different distance matrices, The retrieval precision is defined as:

$$\text{precision}=\frac{\#\{\text{relevant}\cap \text{retrieved}\}}{\#\text{retrieved}}$$ (14)

**Fig 5:**
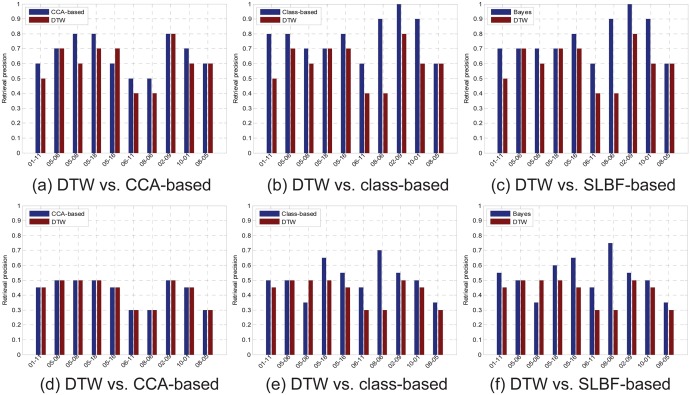
Retrieval precision comparisons between DTW and CCA-base, class-based and Bayes fusion.

We select 10 motion clips from CMU randomly, using each clip as query to retrieve in related motion class. We select 84-dimensinal quaternion descriptor (corresponding to 21 joints of body) as gesture feature, and set *k* clustering centers for each motion clip. We use clustering centers as representative frames to retrieve, the retrieval precision comparisons are shown in Fig 5, the 1^st^ row is precision comparisons of top-10 feedbacks, the 2^nd^ row is precision comparisons based on top-20 feedbacks. The labels correspond to motion capture filenames in CMU database, such as, the 01–11 correspond to “01-11.bvh” in CMU database. The 10 motion semantics are “01–10: playground”, “05–06: dance”, “05–08: dance”, “05–16: dance”, “06–11: basketball”, “08–06: walk”, “02–09: swordplay”, “10–01: soccer”, “08–05: walk/stride”, respectively.

In Fig 5(a), we know the CCA-based distance matrices have better retrieval performance than DTW, with feedback number increased, in Fig 5(d), the CCA-based matrices have almost same retrieval performance as DTW. In fact, in most cases, there always is little correlation influence between two frames, thus, the correlation coefficient usually is *ρ* = 1. In Fig 5(b) and 5(e), we know proposed class-based matrices have also better performance than DTW. In Fig 5(c) and 5(f), obviously, the SLBF-based method have the better performance than CCA-based, class-based and DTW. From all cases comparison results, we know the proposed SLBF matrices is premising.

### 3.2 Retrieval performance analysis

#### 3.2.1 Single semantic clips

For testing the single semantic retrieval performance, we first select 30 motion groups from CMU database, where each group includes 10 motion clips and each clips include only a single semantic, the motion groups are listed in Table 1.

**Table 1:** List of 30 randomly selected motion groups (each group includes 10 motions).

1. climb	6. run/jog	11. salsa dance	16. walk sideways	21. clean1	26. clean2
2. basketball	7. walk terrain	12. swing	17. carry suitcase	22. social dance	27. run turn
3. walk	8. navigate	13. placing ball	18. hop turn	23. play golf	28. walk balancing
4. run	9. dance	14. walk and turn	19. walk around	24. pick up box	29. walk left
5. walk	10. salsa	15. walk right turn	20. jump forward	25. jump	30. weird walks

**Fig 6:**
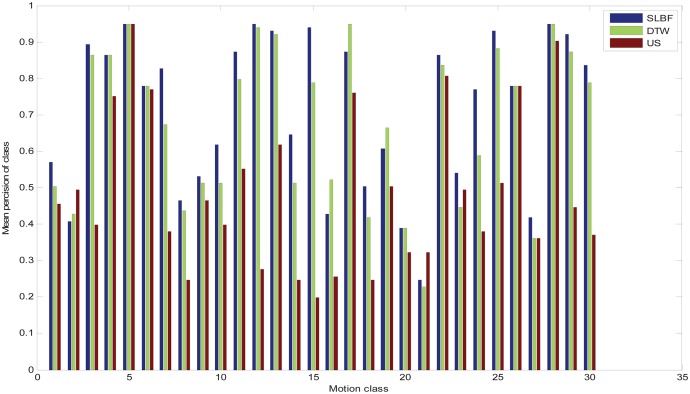
Comparison of retrieval precision of the 30 motion groups.

We calculated the average precision values shown in Fig 6. Here, the 10 motions of each motion class were employed as queries in related motion groups. We select 84-dimensinal quaternion descriptor (corresponding to 21 joints of body) as gesture feature, and set *k* clustering centers for each motion clip. We use clustering centers as representative frames to retrieve, and the precision values of all 10 queries were averaged to obtain the average precision. The retrieval accuracy is evaluated using the precision-recall graph (PR graph):

$$\text{precision}=\frac{\#\{\text{relevant}\cap \text{retrieved}\}}{\#\text{retrieved}}$$ (15)

$$\text{recall}=\frac{\#\{\text{relevant}\cap \text{retrieved}\}}{\#\text{relevant}}$$ (16)

where the #retrieved is the number of retrieved clips and the #relevant is the number of relevant clips.

**Fig 7:**
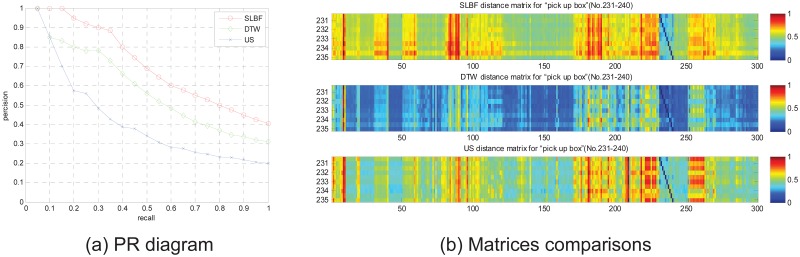
PR diagram and matrices comparisons for motion category “pick up box” on the 30 groups.

In Fig 7, PR comparison diagram and matrices comparisons for motion category “pick up box” on the 30 groups is shown. From the PR comparison results in Fig 7(a), we know SLBF method is superior than DTW and US. At the same time, in Fig 7(b), we compare distance matrixes of 3 different methods, in the field of “No.231-240” (corresponding to 231–240 columns in matrix, the labels 231 to 240 correspond to filenames in CMU database are: “115-01.bvh” to “115-10.bvh”, repetitively.), we see that SLBF-based method has more distinct discrimination from other fields in matrix, that means, SLBF distance metric has better performance than other approaches.

**Fig 8:**
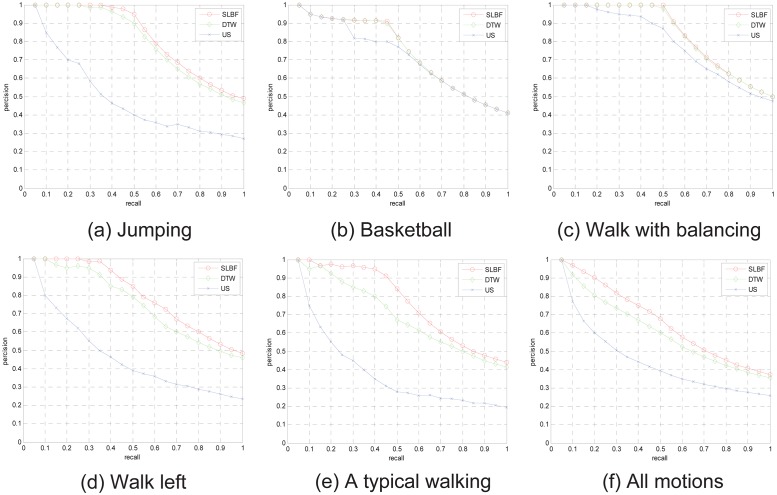
The PR diagram comparisons for motion categories on the 30 groups.

In Fig 8, the more PR comparisons examples are shown. Based on the averaged precision and recall values for the motion classes considered, we see that the above-mentioned three methods perform well, although the proposed method generally performs best. From Fig 8(f), we see that the proposed method performs best when all 30 motion classes are considered. These results confirm that our motion matching algorithm can accurately distinguish different human actions. From the testing results, for simple motions, like running and cleaning, as well as for other motions involving large vertical displacement, such as jumping, the three methods all exhibit a largely equivalent level of performance. However, for more complex motions, such as picking up a box or a typical walking, the proposed method demonstrates significantly better performance than the other methods. Clearly, the proposed method is both suitable for simple and complex motion retrieval.

**Fig 9:**
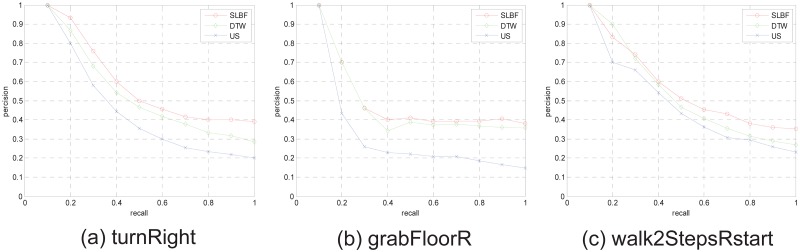
The examples for PR diagram comparisons for motion categories on the whole HDM cut database.

We also test retrieval performance based proposed retrieval frame in HDM database, in this motion database, the long motion sequence has been cut into shorter motion clips, the clips always includes a single action. We select 104-dimensinal quaternion descriptor (corresponding to 26 joints of body) as gesture feature, and use FCM to obtain *k* clustering centers for each motion clip, the longer clips are with more clustering centers. We use clustering centers as representative frames to retrieve. Some PR comparison examples for different motion categories are shown in Fig 9, the 3 different motion categories in HDM database are tested, from retrieval results, we know proposed SLBF method have better performance than DTW and US approach.

#### 3.2.2 Multi-semantic clips

**Fig 10:**
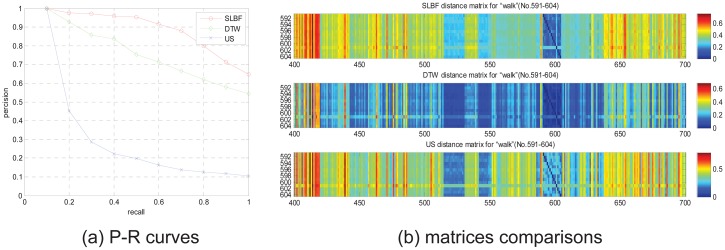
The PR diagram and matrices comparisons for motion subject “walk” on the entire CMU database.

**Fig 11:**
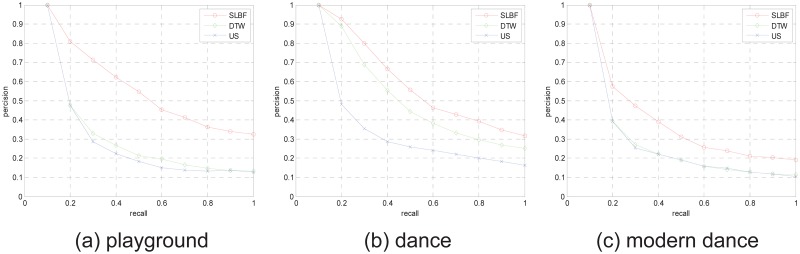
The PR diagram for motion subjects on the whole CMU database.

We also test retrieval performance of SLBF based on long motion sequence that includes more motion semantics. We select 4 groups from CMU database to test retrieval performance, we select 84-dimensinal quaternion descriptor (corresponding to 21 joints of body) as gesture feature, and set *k* clustering centers, use clustering centers as representative frames to retrieve, the test results are shown in Figs 10 and 11.

In Fig 10(a), the PR comparison curves of subject “walk” are shown, we can see SLBF method is superior than other approaches, this subject includes more semantics, such as climb, swing, hang on playground equipment. We use each clips in the subject as query to retrieve in entire CMU motion database. In Fig 10(b), the distance matching matrixes comparison is shown, for easy to observe, only the 400 to 700 columns in distance matrixes are shown. From comparison results, we see, based on SLBF method, the fields (“No.591-604”) distinctly discriminated from other fields in matrix. That means, the proposed SLBF-based method has better discrimination performance than DTW and US approaches. The labels (from 591 to 604) corresponding to filenames in CMU database are: “39-01.bvh” to “39-14.bvh”, repetitively.

In Fig 11, we also give 3 performance comparison examples for multi-semantic clips retrieval of different subjects. In Fig 11(a), the subject “playground” includes clips corresponding to filenames in CMU database are: “01-01.bvh” to “01-14.bvh”, repetitively. This subject includes more semantics, such as climb, swing, hang on playground equipment. In Fig 11(b), the subject “dance” includes clips corresponding to filenames in CMU database are: “49-09.bvh” to “49-17.bvh”, repetitively. This subject includes more semantics, such as lean forward, bring back leg forward, arching arms. In Fig 11(c), the subject “modern dance” includes clips corresponding to filenames in CMU database are: “05-02.bvh” to “05-12.bvh”, repetitively. This subject includes more semantics, such as cartwheel-like start, pirouettes, expressive arms, pirouette, and so on. From retrieval results, we see our method has more retrieval performance than other approaches.

### 3.3 Analysis and discussion

#### 3.3.1 Parameters analysis

As above-mentioned, the number of clustering centers is key parameters to influence over retrieval performance. We have conducted some comparison experiments to obtain the optimal parameters.

**Fig 12:**
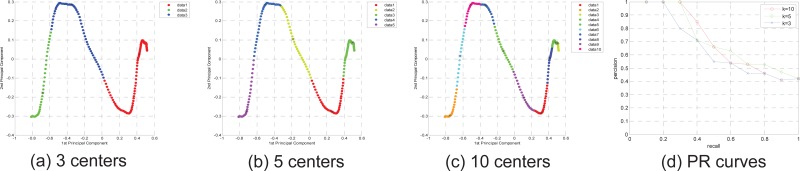
Examples for motion frames clustering using the FCM in HDM database.

Some FCM clustering examples are given in Fig 12, in Fig 12(a), motion clip “turnRight” is clustered using FCM, for easy to observation, we use principle component analysis (PCA) to reduce dimension of gesture descriptor, only 1^st^ principle component and 2^nd^ principle component are shown. When the clustering number is 3, 5 and 10, the clustering results are shown in (a), (b) and (c), respectively. From the clustering examples, we know the bigger *k* value is related to the more original information, however, the retrieval time cost may be higher. We also compare the SLBF retrieval performance using clip “turnRight” based on different *k* value, in Fig 12(d), PR comparisons is given, the results indicate the more centers is with the higher precision. However, we must consider retrieval time cost, for example, when *k* = 10, each query cost time is 2.6s, when *k* = 5, each query cost time is 0.8s, and when *k* = 3, each query cost time is 0.6s, if consider time cost and retrieval precision together, we select *k* = 5. That means, we select clustering coefficient *α* = 45, then all motion clips is clustered according to *α*.

#### 3.3.2 Fails discussion

**Fig 13:**
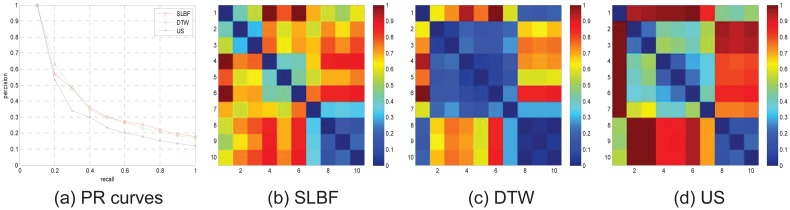
Confusion matrices for motion groups “sneak2StepsRStart” using SLBF-based motion retrieval method, calculated on the HDM05 cut database using FCM clustering feature set and the precision-recall diagram.

For SLBF matching, there are some failing or unsatisfactory retrieval results, for example, in Fig 13, some confusion matrices for motion groups “sneak2StepsRStart” are shown. In Fig 13(a), comparison curves are calculated using SLBF-based motion retrieval frame, calculated on the HDM05 cut database using FCM clustering feature set, the motion group includes 10 motion clips with semantic “sneak2StepsRStart”. In Fig 13(b), SLBF metric matrix is shown, we see those SLBF distances are distinctly discriminated each other even though those motion clips are with same semantic. Although total retrieval performance is still better than DTW and US, the total discrimination performance is unsatisfactory. One reason may be posture changes in this motion group are more complicated, and canonical correlation relationship between frames is hard to find, on the other hand, may be posture changes suddenly, the effect of Bayesian fusion is not ideal. Those problems requires us to conduct further thorough research in the future.

## 4 Conclusions

In this paper, we present a content-based motion retrieval approach. The proposed statistical learning and Bayesian fusion (SLBF) motion similarity matching algorithm first finds representative frames and their corresponding weight values. Based on statistical learning, we obtain possibility models for each motion category. To calculate similarity distances, we utilize two similarity measurement methods, including class-based and CCA-based motion similarity distance measurements. For obtaining further optimized similarity distances, a Bayesian fusion algorithm is employed to update class-based similarity distance predictions using real-time CCA-based motion similarity distance measurements. The proposed retrieval method is tested using motions derived from the CMU and HDM database. The testing results and comparisons with the existing retrieval approaches indicate the promising accuracy and effectiveness of the proposed method.
